# Emergence of a Novel Salmonella enterica Serotype Reading Clonal Group Is Linked to Its Expansion in Commercial Turkey Production, Resulting in Unanticipated Human Illness in North America

**DOI:** 10.1128/mSphere.00056-20

**Published:** 2020-04-15

**Authors:** Elizabeth A. Miller, Ehud Elnekave, Cristian Flores-Figueroa, Abigail Johnson, Ashley Kearney, Jeannette Munoz-Aguayo, Kaitlin A. Tagg, Lorelee Tschetter, Bonnie P. Weber, Celine A. Nadon, Dave Boxrud, Randall S. Singer, Jason P. Folster, Timothy J. Johnson

**Affiliations:** a Department of Veterinary and Biomedical Sciences, College of Veterinary Medicine, University of Minnesota, Saint Paul, Minnesota, USA; b Department of Veterinary Population Medicine, College of Veterinary Medicine, University of Minnesota, Saint Paul, Minnesota, USA; c Mid-Central Research and Outreach Center, University of Minnesota, Willmar, Minnesota, USA; d Public Health Agency of Canada, National Microbiology Laboratory, Winnipeg, Canada; e WDS, Inc., Atlanta, Georgia, USA; f Minnesota Department of Health, Saint Paul, Minnesota, USA; g Division of Foodborne, Waterborne, and Environmental Diseases, Centers for Disease Control and Prevention, Atlanta, Georgia, USA; U.S. Centers for Disease Control and Prevention

**Keywords:** *Salmonella*, clone, genomic, human, outbreak, poultry, turkey

## Abstract

Two separate human outbreaks of Salmonella enterica serotype Reading occurred between 2017 and 2019 in the United States and Canada, and both outbreaks were linked to the consumption of raw turkey products. In this study, a comprehensive genomic investigation was conducted to reconstruct the evolutionary history of *S.* Reading from turkeys and to determine the genomic context of outbreaks involving this infrequently isolated *Salmonella* serotype. A total of 988 isolates of U.S. origin were examined using whole-genome-based approaches, including current and historical isolates from humans, meat, and live food animals. Broadly, isolates clustered into three major clades, with one apparently highly adapted turkey clade. Within the turkey clade, isolates clustered into three subclades, including an “emergent” clade that contained only isolates dated 2016 or later, with many of the isolates from these outbreaks. Genomic differences were identified between emergent and other turkey subclades, suggesting that the apparent success of currently circulating subclades is, in part, attributable to plasmid acquisitions conferring antimicrobial resistance, gain of phage-like sequences with cargo virulence factors, and mutations in systems that may be involved in beta-glucuronidase activity and resistance towards colicins. U.S. and Canadian outbreak isolates were found interspersed throughout the emergent subclade and the other circulating subclade. The emergence of a novel *S*. Reading turkey subclade, coinciding temporally with expansion in commercial turkey production and with U.S. and Canadian human outbreaks, indicates that emergent strains with higher potential for niche success were likely vertically transferred and rapidly disseminated from a common source.

**IMPORTANCE** Increasingly, outbreak investigations involving foodborne pathogens are difficult due to the interconnectedness of food animal production and distribution, and homogeneous nature of industry integration, necessitating high-resolution genomic investigations to determine their basis. Fortunately, surveillance and whole-genome sequencing, combined with the public availability of these data, enable comprehensive queries to determine underlying causes of such outbreaks. Utilizing this pipeline, it was determined that a novel clone of *Salmonella* Reading has emerged that coincided with increased abundance in raw turkey products and two outbreaks of human illness in North America. The rapid dissemination of this highly adapted and conserved clone indicates that it was likely obtained from a common source and rapidly disseminated across turkey production. Key genomic changes may have contributed to its apparent continued success in commercial turkeys and ability to cause illness in humans.

## Abstract (precis)

Increasingly, outbreak investigations involving foodborne pathogens are difficult due to the interconnectedness of food animal production and distribution, and homogeneous nature of industry integration, necessitating high-resolution genomic investigations to determine their basis. Fortunately, surveillance and whole-genome sequencing, combined with the public availability of these data, enable comprehensive queries to determine underlying causes of such outbreaks. Utilizing this pipeline, it was determined that a novel clone of *Salmonella* Reading has emerged that coincided with increased abundance in raw turkey products and two outbreaks of human illness in North America. The rapid dissemination of this highly adapted and conserved clone indicates that it was likely obtained from a common source and rapidly disseminated across turkey production. Key genomic changes may have contributed to its apparent continued success in commercial turkeys and ability to cause illness in humans.

## INTRODUCTION

Salmonella enterica subsp. *enterica* derived from poultry meat serves as a primary cause of salmonellosis infections in humans within the United States and worldwide (1, 2). Among the more than 2,500 serotypes that have been identified thus far, only a handful of them consistently top the list as those causing the majority of cases of human illness. Estimates on human salmonellosis cases from poultry in the United States vary, depending on the method used, from 10 to 29%, and the estimate for cases specifically from turkeys numbers 5.5% (3, 4).

*S*. Reading is a serotype of S. enterica subsp. *enterica* first identified in 1916 from a water supply in Reading, England (5), and subsequently identified in various animal hosts, including poultry (6–10). Human outbreaks due to *S*. Reading historically have been relatively infrequent. In 1956 to 1957, an outbreak involving *S*. Reading occurred in the United States, sickening 325 people across multiple states (11). In 2008, 30 persons were involved in an outbreak linked to iceberg lettuce in Finland (12). In 2014 to 2015, an outbreak of unknown origin was described, with 31 confirmed cases in Canada involving persons of Mediterranean descent (13).

Commercial turkey production is commonly identified as a primary reservoir of *S*. Reading (2, 14–17). Given its low isolation frequency, relatively little is known about the biology of *S*. Reading compared with other serotypes. With that said, *S*. Reading has been shown to have enhanced ability to form biofilms under stress conditions (18) and has been isolated from produce (19). Multidrug resistance phenotypes, including resistance towards third-generation cephalosporins, also appear to be common in *S*. Reading strains, including those in dairy cows and beef feedlot cattle (20–22).

Two separate, large outbreaks of *S*. Reading were recently reported in North America. In the United States, the Centers for Disease Control and Prevention declared an outbreak from November 2017 through March 2019 (23), although human cases of salmonellosis due to *S*. Reading have continued (as of January 2020). The outbreak was linked to live turkeys and raw turkey products, but no single source product or company was attributed to the entire outbreak. This outbreak resulted in 358 illnesses, 133 hospitalizations, and 1 death across 42 states. In Canada, a separate multiprovince outbreak was declared in October 2018 by the Public Health Agency of Canada, with a final report in February 2020 of 130 identified cases (24).

Given the widespread nature of these recent North American *S*. Reading outbreaks, there is a pressing need to better understand the ecology and evolution of this foodborne pathogen within suspected animal reservoirs. As such, the purpose of this study was to perform a comprehensive genomic investigation to reconstruct the evolutionary history of *S*. Reading and to determine whether underlying genomic changes within *S*. Reading correlated with outbreaks involving this rarely isolated *Salmonella* serotype.

## RESULTS

### *S.* Reading isolates cluster phylogenetically by host source.

**FIG 1:**
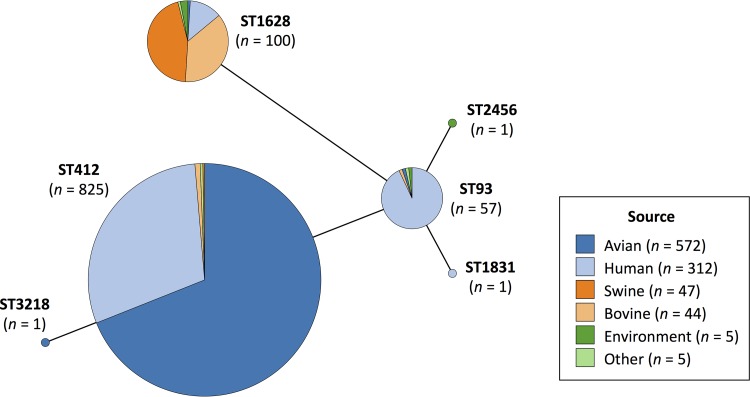
Minimum spanning tree of STs using the Achtman seven-gene MLST scheme for 985 *S*. Reading isolates. Three isolates (swine-, chicken-, and human-source isolates) are not included because their STs could not be determined. The tree is colored based on the isolate host source.

Using assembled sequences (*n* = 988) from human illness, meat products, live animals, and environmental sources, isolates were first assigned to seven-gene multilocus sequence types (MLSTs) using the scheme from the PubMLST website (https://pubmlst.org) (25). Based on this scheme, six sequence types (STs) were identified with three dominating: one containing primarily turkey-source and human-source isolates (ST412; 83.5% of isolates), one containing primarily swine/bovine-source and human-source isolates (ST1628; 10.1% of isolates), and one containing primarily human-source isolates (ST93; 5.8% of isolates) (Fig. 1). Animal host source was strongly correlated with ST, with 99.6% (564/566) of total turkey-source isolates belonging to ST412 and 93.8% (45/48) and 84.1% (37/44) of swine-source and bovine-source isolates, respectively, belonging to ST1628. To rule out temporal bias in the clustering of same host-source isolates by ST, isolates were also characterized based on year of isolation using the same ST scheme (see Fig. S1 posted at https://doi.org/10.6084/m9.figshare.11966550). This demonstrated evenness with regard to isolation date across the major STs.

Core genome MLST (cgMLST) profiles based upon 3,002 loci were then identified for all isolates, allowing for up to either two allelic differences (see Fig. S2A posted at the above URL) or five allelic differences (see Fig. S2B posted at the above URL). In all analyses, there was clear and consistent separation based upon animal host source, separating isolates into three major groups.

**FIG 2:**
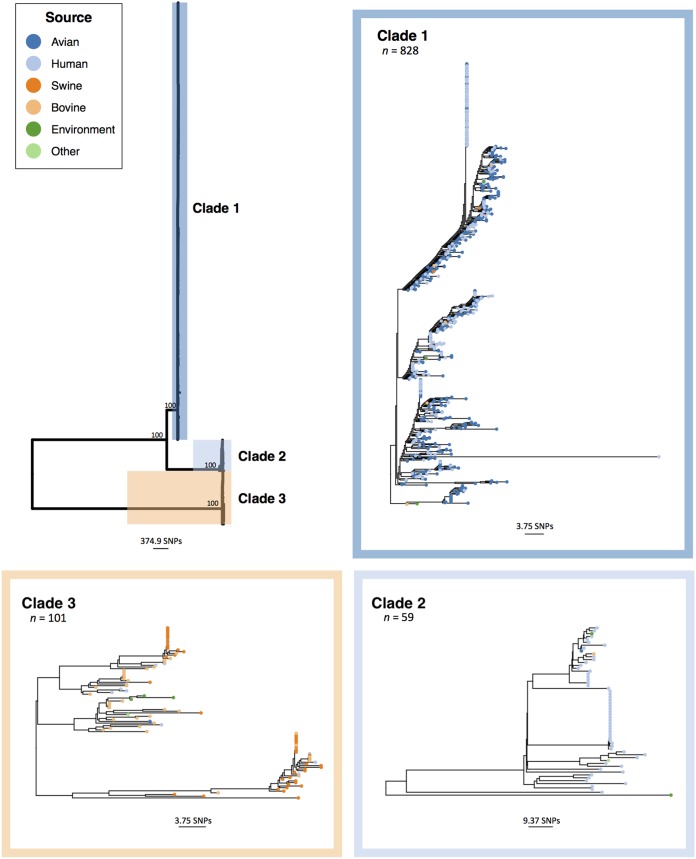
Midpoint-rooted phylogenetic tree of *S*. Reading isolates (*n* = 988) based on core SNPs in nonrecombinant genome regions. All isolates fell into one of three clades: clade 1 (dark blue; primarily turkey- and human-source isolates), clade 2 (light blue; primarily human-source isolates), and clade 3 (orange; primarily swine- and bovine-source isolates). Bootstrap values are shown at the branches differentiating between clades. To allow for a finer-scale view of clade topology, insets show each clade independently (note the difference in scale bars). The color of the circles at the tips indicates the isolate host source.

To gain further resolution, a whole-genome core single nucleotide polymorphism (SNP)-based phylogenetic tree was constructed for all isolates (Fig. 2; see Fig. S3 posted at the above URL for a greater resolution tree including all bootstrap values). The resulting tree contained 11,086 core SNPs and resolved isolates into three primary clades (designated clades 1 to 3), corresponding to MLST and cgMLST results. Clade 1 (*n* = 828) was comprised mainly of turkey-source and human-source isolates, and all but one turkey-source isolate fell within this clade. Clade 2 (*n* = 59) was primarily human-source isolates. Clade 3 (*n* = 101) contained mainly swine-source and bovine-source isolates, with 95.8% (46/48) and 84.1% (37/44) of total swine-source and bovine-source isolates falling within this clade, respectively. Average core SNP distances were investigated between clades (see Table S1 posted at https://doi.org/10.6084/m9.figshare.11966550), revealing that clades 1 and 2 were more similar to one another (mean core SNP difference, 1,638.72 ± 8.49) than clades 1 and 3 (8,165.04 ± 10.91) or clades 2 and 3 (9,246.30 ± 12.72). Additionally, mean SNP differences for isolates within clade 1 (7.72 ± 5.61) were lower than those within clade 2 (59.23 ± 44.10) or clade 3 (32.87 ± 16.84). To confirm that these results were not due to different sample sizes between clades, average core SNP distances were recalculated on a random subsample of each clade (see Table S1 posted at the above URL).

Genome sizes also varied between the three clades, with clade 2 containing the smallest genomes (median, 4.53 ± 0.095 Mb), which were on average 114.50 kb smaller than clade 1 genomes (median, 4.64 ± 0.050 Mb) and 396.25 kb smaller than clade 3 genomes (median, 4.92 ± 0.10 Mb) (see Fig. S4 posted at the above URL).

A pan-genome approach was then used to investigate specific genomic differences between isolates from clades 1 and 3, representing the majority of isolates from turkey and swine/bovine sources, respectively. A total of 11,366 gene clusters were identified across all 988 isolates, with 3,246 (28.6%) present in 100% of isolates (i.e., the “core” genes). Using a cutoff requirement of 100% prevalence versus 0% prevalence in the two populations, a total of 225 gene clusters were identified as unique to clade 1, and 180 gene clusters were unique to clade 3 (see Dataset S1 posted at https://doi.org/10.6084/m9.figshare.11966550). Clade 1 isolates had 15 unique fimbrial system component genes clustered across three systems, including *yadKLMNV*, *yehABCD*, and a novel K88-like fimbrial system, all of which were inserted in separate genomic locations with genes for each respective system clustered together. Clade 1 isolates also uniquely possessed *prgHIK* and *orgAB*, which are components of the *Salmonella* pathogenicity-associated island SPI-1 (26), genes annotated as cytolethal distending toxin *cdtAB*, and several prophage-like elements. Conversely, clade 3 isolates possessed a number of unique fimbria-like and prophage-like elements compared to those from clade 1. Also unique to clade 3 isolates were systems predicted to be involved in type I restriction modification, phosphotransferase activity, and CRISPR/Cas activity.

### A recently emerged clade exists among turkey-source *S*. Reading isolates.

**FIG 3:**
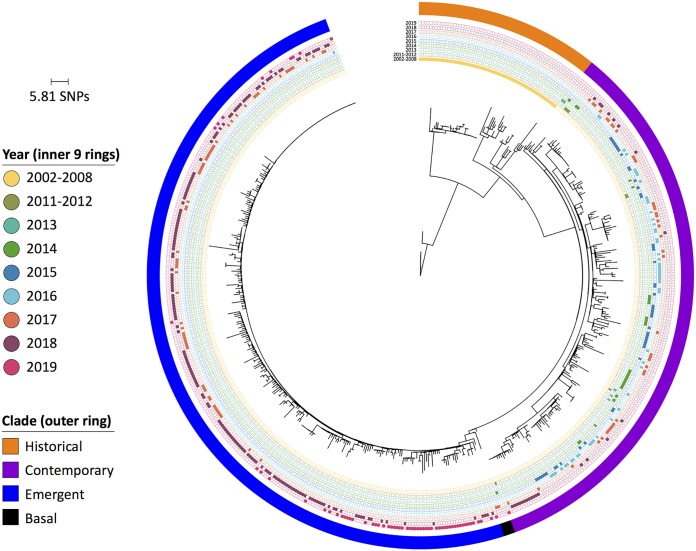
Phylogenetic tree of turkey-source *S*. Reading isolates (*n* = 565) based on core SNPs in nonrecombinant genome regions. The majority of isolates were grouped based on clustering and isolation year into three subclades shown in the outer ring. The inner nine rings show years of isolation, with filled circles depicting the year for an individual isolate. The tree is rooted with an isolate collected in 2002 (SRR1195634).

The turkey-source isolates from clade 1 were then examined alone to gain further insight towards their evolution over time. All of these isolates (*n* = 565), except one, belonged to ST412 and were examined at higher resolution using a core SNP-based phylogenetic tree (Fig. 3). The phylogenetic tree contained 1,093 informative variant sites, and from this, three major subclades were designated based upon tree clustering and dates of isolation. The “historical” subclade (orange subclade in Fig. 3; *n* = 65) contained isolates dating 1999 to 2008. The “contemporary” subclade (purple subclade in Fig. 3; *n* = 201) contained isolates dating 2009 to 2019, with the majority from 2009 to 2016. Finally, the “emergent” subclade (blue subclade in Fig. 3; *n* = 295) contained isolates all dating 2017 to 2019, except for one from 2016. Four isolates were not assigned to a specific subclade due to their intermediate location between the contemporary and emergent subclades (black “basal” subclade in Fig. 3).

**FIG 4:**
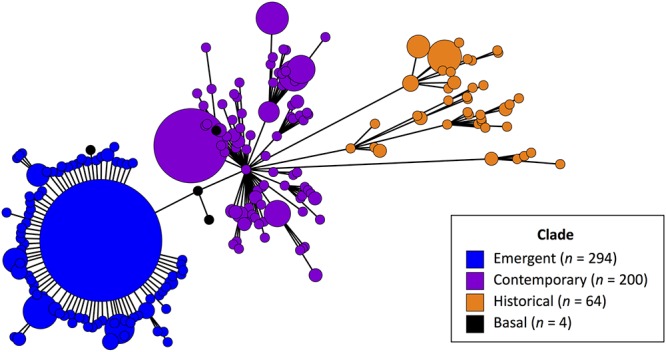
Minimum spanning tree of turkey-source isolates (*n* = 562) using the core genome sequence typing (cgMLST) scheme allowing for up to two allelic differences. Three isolates are not included because their cgMLST profiles could not be determined. Tree colors are based on core SNP-based phylogenetic tree subclade designations (see Fig. 3). Four isolates not assigned to a specific subclade are classified as basal to the emergent subclade (black color).

The same three-subclade structure was also observed in a minimum spanning tree from cgMLST data allowing for up to two allelic differences (Fig. 4), where isolates clearly separated by subclade designation (historical, contemporary, and emergent) and 57.6% of all isolates in the emergent subclade were of the same cgMLST profile. A phylogenetic tree constructed from core genome SNPs and a dendrogram based on hierarchical clustering of all pan-genome genes also showed isolates clustered into the same three subclades (see Fig. S5 posted at the above URL).

Based upon average core SNP distances (Table 1), the emergent and contemporary subclades were more similar to each other (mean core SNP difference, 14.35 ± 3.08) than emergent versus historical subclade (39.95 ± 11.38) or contemporary versus historical subclades (42.58 ± 11.59). Within subclades, emergent subclade isolates were more similar to each other (4.67 ± 2.13) than were isolates from the contemporary (10.92 ± 3.88) or historical (33.63 ± 18.37) subclade.

**TABLE 1:** Comparison of mean core SNP differences between unique core SNP profiles in the same and different turkey-only phylogenetic subclades

Subclade comparison	SNP difference		
	Mean ± SD	Minimum	Maximum
All profiles^*a*^			
Overall	16.74 ± 14.04	1	78
Emergent	4.67 ± 2.13	1	18
Contemporary	10.92 ± 3.88	1	23
Historical	33.63 ± 18.37	1	73
Emergent vs contemporary	14.35 ± 3.08	6	29
Emergent vs historical	39.95 ± 11.38	23	78
Contemporary vs historical	42.58 ± 11.59	21	77

Random profile subset^*b*^			
Overall	27.18 ± 17.51	1	76
Emergent	5.27 ± 1.95	1	12
Contemporary	10.89 ± 4.13	1	22
Historical	33.63 ± 18.37	1	73
Emergent vs contemporary	14.61 ± 3.19	8	24
Emergent vs historical	40.18 ± 11.41	24	74
Contemporary vs historical	41.61 ± 11.60	22	76

a The numbers of unique core SNP profiles were as follows: *n* = 44 for the historical subclade, *n* = 151 for the contemporary subclade, and *n* = 200 for the emergent subclade.

b For the random profile subset, there were 44 unique core SNP profiles from each subclade.

### Small plasmids and associated resistance genes define differences between turkey-source clades.

**FIG 5:**
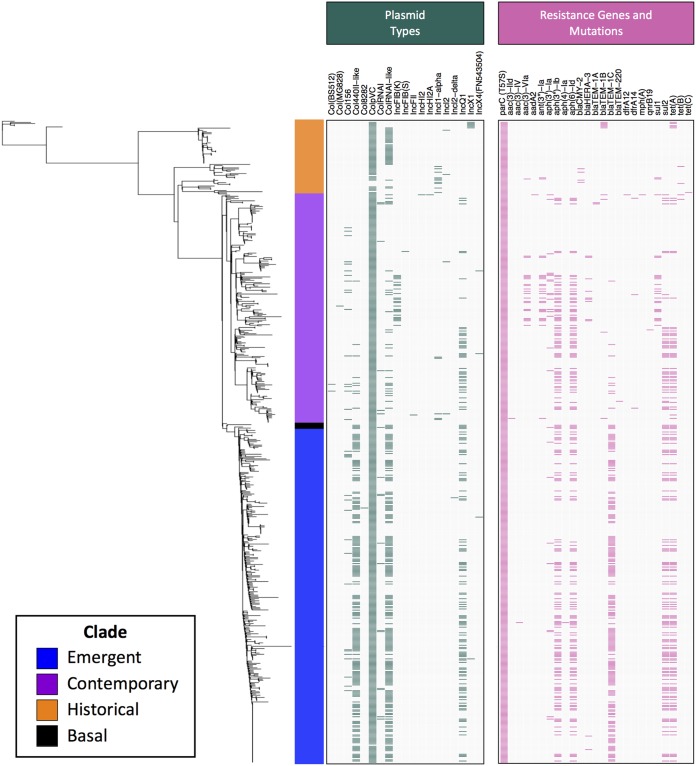
Heatmap displaying the presence of plasmid replicons (dark green) and genes and mutations conferring antimicrobial resistance (pink) across clade 1 turkey-source isolates.

All clade 1 turkey-source isolates were examined for their possession of genes and mutations known to confer antimicrobial resistance and plasmid replicons known among Gram-negative bacteria (Fig. 5). When overlaid on the SNP-based phylogenetic tree, several patterns emerged. First, nearly all isolates contained a T57S mutation in *parC* and the ColpVC plasmid replicon. An IncQ1 plasmid replicon was found in 20% (41/201) and 33% (98/295) of isolates belonging to the contemporary and emergent subclades, respectively. The possession of this plasmid replicon was significantly associated with possession of *sul2*, *tet*(A), *strA* [*aph(3*′′*)-Ib*], and *strB* [*aph*(*6)-Id*] genes conferring the classical SSuT phenotype (see Table S2 posted at https://doi.org/10.6084/m9.figshare.11966550; all pairwise Fisher’s exact test Benjamini-Hochberg (BH)-adjusted *P* values < 0.05). Possession of these traits were found throughout the emergent subclade, with some evidence of trait loss scattered infrequently. In contrast, isolates possessing these traits in the contemporary subclade were found clustered in one half of the subclade and were absent from the other half.

Isolates belonging to the emergent subclade also frequently possessed co-occurring ColRNAI-like and Col440II-like plasmid replicons, present in 61% (181/295) and 65% (191/295) of isolates within this subclade, respectively. Isolates in the emergent subclade were more than 20 times more likely to possess both replicons compared to isolates in the historical and contemporary subclades (Fisher’s exact test: odds ratio = 0.022, *P* value < 0.05). Possession of these replicons was also significantly associated with possession of the beta-lactam resistance gene, *bla*_TEM-1C_ (see Table S2 posted at the URL mentioned above; all pairwise Fisher’s exact test BH-adjusted *P* values < 0.05).

**FIG 6:**
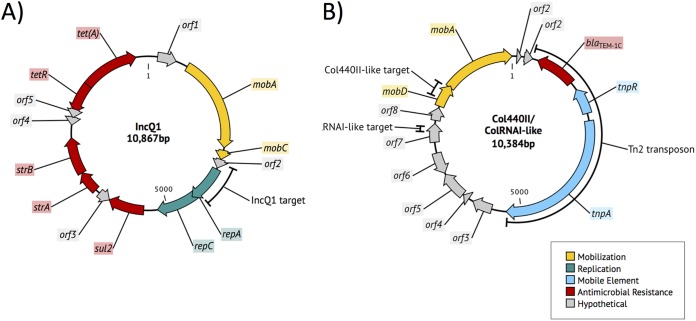
Circular genetic maps of IncQ1 (A) and Col440II/ColRNAI-like (B) plasmids. Arrows indicate predicted genes and the direction of transcription and are colored to indicate predicted functional category.

Complete sequences of these highly conserved plasmids belonging to IncQ1 and Col440II/ColRNAI-like replicon types were identified and annotated from a representative turkey-source isolate (Fig. 6). The IncQ1 replicon and *sul2-strAB-tetAR* genes were colocalized within a 10,867-bp mobilizable plasmid containing *mobAC*. The Col440II- and ColRNAI-like replicons were found on a 10,384-bp mobilizable plasmid containing *mobAD* and *bla*_TEM-1C_ adjacent to a Tn*2* transposon.

To better understand the emergence of this Col440II/ColRNAI plasmid variant, all surveillance data from the European Nucleotide Archive (ENA) and NCBI Short Read Archive (SRA) databases (until December 2016) were searched for a 282-bp region of the Col440II-like replicon (see Fig. S6 posted at https://doi.org/10.6084/m9.figshare.11966550). The first available sequence of the replicon was identified in S. enterica in 2000, but it did not carry *bla*_TEM-1C_. The first detection of this plasmid replicon carrying *bla*_TEM-1C_ was from a turkey-source *S*. Hadar isolate in 2007, and the appearance of this plasmid replicon in *S*. Hadar coincided with subsequent foodborne outbreaks implicating live poultry or poultry products (27, 28), with isolates from those outbreaks containing highly similar plasmids (nucleotide blast of draft assemblies; data not shown). The first detection of this plasmid replicon, including *bla*_TEM-1C_, in *S*. Reading was from a turkey-source isolate in 2014.

### Pan-genome-wide association analysis suggests that clusters of bacteriophage-associated genes and other elements were gained and lost over time.

Comparison of average genome sizes between subclades showed an increase in size from the historical subclade (median, 4.58 ± 0.051 Mb) to the contemporary subclade (median, 4.66 ± 0.046 Mb) and a subsequent decrease in size to the emergent subclade (median, 4.63 ± 0.016 Mb) (see Fig. S4 posted at the above URL). A pan-genome analysis was used to identify specific genes contributing to this shift in genome size between subclades. A total of 6,747 gene clusters were produced, of which 3,763 (56%) were core genes. Of the 2,984 accessory genes, the majority (79%) were found in less than 15% of isolates (see Fig. S7 posted at the above URL).

**FIG 7:**
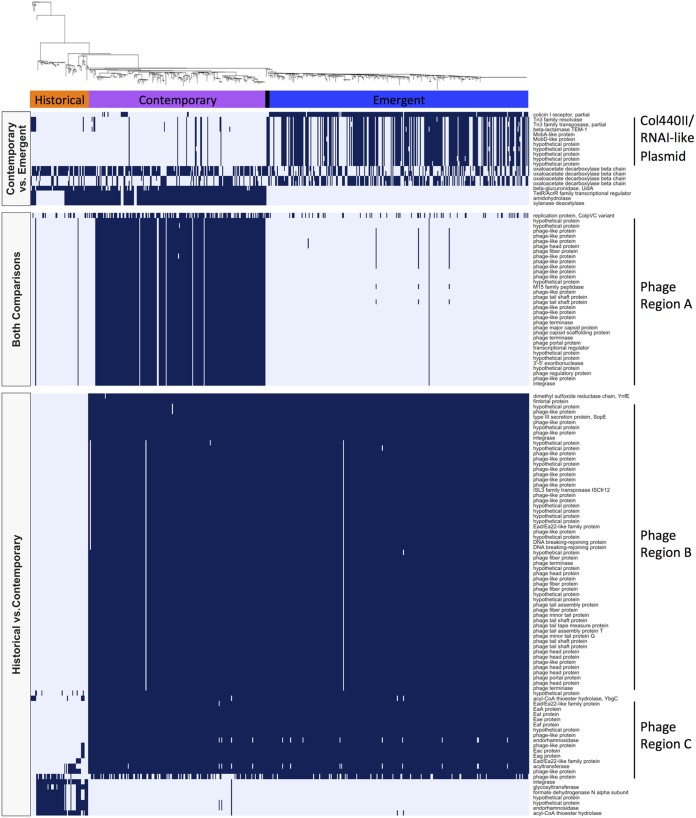
Heatmap displaying the presence (dark blue) and absence (light blue) of genes with significant associations to the historical, contemporary, and/or emergent subclades. Left-hand side labels group genes based on the comparison they were identified in: historical versus contemporary, contemporary versus emergent, or both comparisons. Right-hand side labels denote genes that clustered into a single region of the *S*. Reading genome.

**FIG 8:**
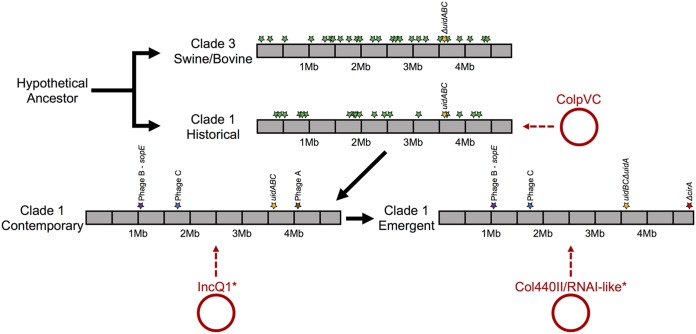
Genetic changes leading from the hypothetical ancestor of *S*. Reading through the current emergent turkey-source clonal group. Green stars indicate unique genomic islands differing between clades 1 and 3. Purple, blue, and brown stars indicate insertions within clade 1 contemporary and emergent isolates relative to historical subclade isolates. The gold star indicates insertion of *uidABC*-like region in clade 1 isolates, where the *uidA*-like gene was subsequently truncated in emergent subclade isolates. The red star indicates a truncation of the *cirA* gene in clade 1 emergent subclade isolates. Plasmid acquisitions are denoted by circles and dashed arrows. Note that IncQ1 and Col440II/RNAI-like plasmids are found in some other clades but become dominant in the denoted subclades.

Pan-genome-wide association analysis identified 134 genes with significantly differential prevalence between the historical, contemporary, and emergent subclades (Fig. 7; see Dataset S2 posted at https://doi.org/10.6084/m9.figshare.11966550). A large collection of genes primarily encoding bacteriophage-related proteins was absent from the majority of both historical and emergent isolates (<2.5%) but was found in most contemporary isolates (93%) (phage region A in Fig. 7). Based on annotations of the representative genome assembly, SRR2407706, all of these genes were clustered in a single region of the *S*. Reading genome (Fig. 8; see Fig. S8 posted at the above URL), and the majority were homologous to genes from bacteriophages HP1 and HP2. Two separate collections of bacteriophage-related genes were absent from all historical subclade isolates, but present in more than 99% of contemporary and emergent subclade isolates (phage regions B and C in Fig. 7). Both gene clusters could be mapped to separate regions of the *S*. Reading genome (Fig. 8; see Fig. S8 posted at the above URL), with phage region B genes homologous to genes primarily found in lambda phages GIFSY-1 and GIFSY-2 and phage region C genes homologous to a range of *Enterobacterium*-specific phages. Of particular note, phage region B included the bacterial virulence-associated gene *sopE* encoding a type III secretion protein effector, which was surrounded by genes encoding phage tail and fiber proteins and an IS*L3* family transposase.

Of the 13 genes significantly associated with the emergent subclade, 10 were identified as part of the Col440II/RNAI-like plasmid. These 10 genes included genes encoding TEM-1C beta-lactamase, Tn*2* transposase and resolvase, mobilization proteins A and D, and five hypothetical proteins. The Col440II-like replicon was significantly more common in isolates from the emergent subclade than in isolates from either the historical or contemporary subclades (all pairwise Fisher’s exact test BH-adjusted *P* values < 0.05) (see Tables S3 and S4 posted at the above URL). Additionally, *cirA*, which encodes a colicin Ia/b receptor, was identified intact in 93.5% of isolates in the contemporary and historical subclades but was disrupted in the majority (96.9%) of isolates from the emergent subclade due to a frameshift insertion of cytosine at position 680. In some of the contemporary subclade isolates, amino acids 47 to 69 of *cirA* were truncated, representing a distinct disruption of CirA compared to the emergent isolates. Similarly, a full-length *uidA*-like gene, which is predicted to encode a beta-glucuronidase enzyme, was present in 89.8% of contemporary and historical subclade isolates but truncated in all emergent subclade isolates. Interestingly, *uidABC* was also found to be absent from clade 3 isolates in unique fashion compared to clade 1 emergent subclade isolates.

### Time-scaled phylogenetic analysis.

A time-scaled phylogeny of turkey-source sequences (*n* = 398 after removal of duplicated sequences) was reconstructed using a general time reversible (GTR) nucleotide substitution model, an uncorrelated lognormal relaxed molecular clock, and a constant growth coalescent model (see Fig. S9 at https://doi.org/10.6084/m9.figshare.11966550). The model predicted an evolutionary rate of 4.14 × 10^−7^ substitutions/site/year (95% higher posterior density [HPD_95_] = 3.60 × 10^−7^ to 4.77 × 10^−7^) and time to most recent common ancestor (TMRCA) for clade 1 was dated to 1984 (1975 to 1992). The branching of the contemporary and emergent subclades was dated to 1997 (1994 to 1997) with the emergent subclade arising in 2015 (2014 to 2016).

### North American *S*. Reading outbreak isolates cluster with both contemporary and emergent subclade turkey-source isolates.

To investigate the two recent North American *S*. Reading outbreaks in the context of turkey-source *S*. Reading strains, a core SNP-based phylogenetic tree was constructed for all clade 1 turkey-source isolates (*n* = 565) and human-source isolates identified as part of the 2017 − 2019 *S*. Reading outbreaks in the United States (*n* = 139) and Canada (*n* = 111) (see Fig. S10 posted at the above URL). Outbreak isolates from both countries were found clustered with turkey-source isolates from both the contemporary and emergent subclades. Specifically, for the U.S. outbreak isolates, 29.5% (41/139) of isolates clustered with the contemporary subclade and 69.1% (96/139) with the emergent subclade. For Canadian outbreak isolates, the distribution was more balanced between subclades, with 47.7% (53/111) clustering with the contemporary subclade and 52.3% (58/111) with the emergent subclade. A subset of both U.S. and Canadian outbreak isolates shared identical core SNP profiles with some turkey-source isolates. In particular, one prevalent SNP profile was found in 96 isolates, including 56 turkey-source isolates, 28 U.S. outbreak isolates, and 12 Canadian outbreak isolates. Mining of CDC and Minnesota of Department of Health data suggests an increase in *S*. Reading starting in 2014 involving clade 1 contemporary subclade isolates. Increases in *S*. Reading cases were further amplified by clade 1 emergent subclade isolates starting in 2016, which increased substantially in relative proportion in 2017 and 2018 (Table 2).

**TABLE 2:** Human cases of *S*. Reading compared with percentage of human-source isolates used in this study that cluster with turkey-source isolates^*a*^

Year	No. of cases		% cases associated with the following subclade:	
	CDC	MNDH	Contemporary	Emergent
2008	46	3	ND	ND
2009	53	3	ND	ND
2010	33	1	ND	ND
2011	42	1	ND	ND
2012	58	3	0	0
2013	55	2	0	0
2014	104	4	82	0
2015	139	7	88	0
2016	221	7	59	2
2017	ND	13	44	29
2018	ND	21	23	63

a Human cases of *S*. Reading reported by the CDC and Minnesota Department of Health (MNDH), compared with percentage of human-source isolates used in this study that cluster with turkey-source contemporary or emergent subclade isolates. ND, no human case data available.

## DISCUSSION

Multiple outbreaks of *S*. Reading in North America prompted an investigation of the microevolution of this serotype, as human-associated outbreaks due to *S*. Reading are infrequently reported compared with other common serotypes. Very clear separation was observed between turkey-source and bovine/swine-source *S*. Reading isolates, accompanied by large whole-genome SNP differences and numerous genomic island differences. This clear separation without intermediate isolates between the two clades (clade 1 versus clade 3) suggests that current clades represent distinct lineages associated with turkey versus bovine/swine hosts. Within clade 1, a time-scaled phylogeny reconstruction demonstrated the diversification of subclade branches with estimated node ages that align with the current North American outbreaks. In addition, these analyses estimated an evolutionary rate of 4.14 × 10^−7^ substitutions/site/year, which corresponds to a change of two SNPs per year. The constant population growth selected here may reflect the early stage of this clonal group’s spread. The data indicate two distinct expansions of *S*. Reading. First, the contemporary subclade began the expansion in 2014 with an increased number of human cases compared to previous years (Table 2). In 2017, the number of human cases again expanded with the surfacing of the emergent subclade, coinciding with multiple outbreaks declared in the United States and Canada.

Genetic diversity within clade 1 was lowest compared to all clades studied here, and genetic diversity within the clade 1 emergent subclade isolates was extremely low. This, combined with dates of isolation, points to the recent emergence of a new clonal group of *S*. Reading, which was estimated to emerge in 2015 (HPD_95,_ 2014 to 2016) based on the time-scaled phylogeny reconstruction. This emergence coincides with large outbreaks in North America linked to contaminated turkey products, prompting the question of why this clonal group and associated serotype have become more successful. The overall genetic differences between the turkey subclades were subtle yet may provide important clues highlighting the success of strains within the contemporary and emergent subclades. One distinguishing feature of the emergent strains, and contemporary subsets of the circulating subclade, was the presence of mobilizable IncQ1 and Col440II/ColRNAI-like small plasmids. Collectively, these plasmids encode resistance towards ampicillin, streptomycin, sulfamethoxazole, and tetracycline. IncQ1 plasmids are broad-host-range, highly mobilizable plasmids capable of residing in a variety of Gram-negative bacterial species (29). Similar conformations of this plasmid conferring the same SSuT resistance profile have been identified in *Salmonella* Typhimurium in Italy (30). While the presence of these two plasmids appears to be a marker of evolution of the subclade, they apparently have been frequently lost by isolates in the emergent subclade. There was no association between isolate host source and apparent plasmid loss (i.e., human- versus turkey-source isolates), indicating that plasmid loss is not a function of selective pressure in a particular environment but instead a function of genetic gain followed by plasmid instability or dispensability.

There was an overall genome size gain between the historical to contemporary/emergent subclade isolates within clade 1. This was primarily due to acquisition of several phage-like elements within the chromosome. Acquisition of a lambda-like prophage-like element was accompanied by accessory carriage of *sopE* into the contemporary and emergent subclades (Fig. 8). All clade 1 turkey strains carried the canonical version of *Salmonella* pathogenicity-associated island, SPI-1. SopE, along with SopE2, are guanine nucleotide exchange effector molecules for the type III secretion system encoded by SPI-1 (31). Together, these two molecules are able to act differentially on the RhoGTPase signaling cascade and may promote enhanced inflammatory function. SopE has also been shown to enhance murine colitis (32). SopE has previously been identified on a P2 family phage-like element in *S.* Typhimurium (33) and was associated with persistent epidemic strains in humans and animals. SopE has also been shown to reside on diverse phage types, including lambda-like phage in *Salmonella* Gallinarum, Enteritidis, Hadar, and Dublin (34), and was more common in the most common human serotypes in England (35). Therefore, the acquisition of SopE by contemporary and emergent subclade isolates may represent an advantage for their persistence and virulence.

Two gene disruptions were notable between the emergent and contemporary isolates of clade 1. First, emergent subclade isolates possessed a frameshift insertion of cytosine at position 680 in the *cirA* gene, resulting in a predicted frameshift that was uniform across emergent isolates. Additionally, a portion of the contemporary subclade isolates possessed a truncation of *cirA* that was independent of the mutation identified in emergent isolates. CirA is a catecholate siderophore receptor that also serves as the receptor for colicin ColIb, a pore-forming toxin produced by some Escherichia coli and *Salmonella* as a competitive exclusion mechanism (36). ColIb production has been shown to favor producers during competition with ColIb-sensitive strains lacking the plasmid that encodes this system (37). However, mutations in *cirA* have rendered ColIb-sensitive strains resistant to the killing effects of ColIb (38). Furthermore, ColIb is commonly found to reside on IncI1 plasmids, which are ubiquitous among members of the family *Enterobacteriaceae* found in commercial turkeys (39, 40). Therefore, it is plausible that disruption of *cirA* in emergent subclade isolates provides a competitive advantage in the gastrointestinal tract against challenging ColIb-positive bacteria. Because disruption of this gene was observed convergently in the contemporary and emergent subclades, it warrants further study.

A second gene disruption identified among emergent subclade isolates that was not present in contemporary or historical isolates was a deletion of a *uidA*-like sequence accompanied by deletion of an adjacent gene predicted to encode peptidoglycan deacetylase, PgdA. This region was intact in contemporary and historical isolates. Interestingly, clade 3 isolates were missing the entire *uidABC* region but retained *pgdA*. The presence of *uidABC* was sought among other phylogenetically proximal *Salmonella* serotypes (41) and was universally present, agreeing with previous studies identifying *Salmonella* clade-specific beta-glucuronidase activity (42). Together, this indicates that the *uidABC* system was ancestrally intact and subsequently truncated/deleted independently in clade 1 emergent and clade 3 isolates. The *uidABC* operon encodes enzymes capable of breaking down glucuronidated ligands, freeing them up as a bacterial nutrient source (43). This is typically viewed as a competitive advantage for gut bacteria. However, because these systems were convergently inactivated in two distinct host-adapted clades of *S*. Reading and beta-glucuronidase systems are known to have a diverse array of functional effects in the gut (44), the possible role of inactivation of this system as a fitness benefit deserves further study.

This study was prompted by two large outbreaks of *S*. Reading in North America linked to the consumption of raw turkey products (23, 24). Our analyses indicate that these outbreaks coincide with the emergence of a novel successful clonal group of *S*. Reading in North America and dramatically increased rates of isolation of *S*. Reading in commercial turkey production, independent of company or geographical region. Given these facts, it is quite likely that the introduction of this clonal group occurred in commercial turkey production rapidly and uniformly. The most parsimonious explanation is that it was introduced vertically from a common source, likely through supply birds at the top of the genetic breeding pyramid. Interestingly, the emergence of this clonal group coincides with an outbreak of highly pathogenic avian influenza in 2015 that decimated turkey breeder supplies in the upper Midwestern United States (45). Thus, the emergence of this clonal group, combined with rapid repopulation efforts in the turkey industry, may have further contributed to its rapid spread. The microevolution of *S*. Reading in turkeys towards the emergent clade has apparently provided it with evolutionary advantages for success in the growing turkey, the turkey barn environment, and/or the human host. Limitations exist in this study, since it used retrospective samples from multiple sources with sometimes inconsistent methods of isolation and missing metadata. Therefore, while it is impossible at this time to pinpoint the precise source, this study highlights the power and utility of high-resolution genomics for better understanding the ecology and evolution of outbreaks of foodborne pathogens.

## MATERIALS AND METHODS

### Sample collection and DNA sequencing.

Thirty-two isolates from this study were collected from commercial turkey production facilities in the United States between October 2016 and October 2018. Samples represent 32 unique premises within multiple turkey-producing companies. Samples were collected by boot sock sampling, environmental swabbing, fluff sampling, or cecal sampling. Enrichments were performed for *Salmonella* by primary enrichment of 1 g sample content in 9 ml in tetrathionate broth overnight with shaking at 42°C, followed by streaking of the primary enrichment onto XLD agar and incubation overnight at 37°C. Serotyping was performed on isolates following a standard protocol (46). DNA was extracted from cultures using the Qiagen DNeasy kit (Valencia, CA) following the manufacturer’s instructions. Genomic DNA libraries were created using the Nextera XT library preparation kit and Nextera XT index kit v2 (Illumina, San Diego, CA), and sequencing was performed using 2x250-bp dual-index runs on an Illumina MiSeq at the University of Minnesota Mid-Central Research and Outreach Center (Willmar, MN).

### Study population for phylogenomic analysis.

A search of NCBI’s Short Read Archive (SRA) was conducted for all available raw sequencing data of isolates annotated as Salmonella enterica subsp. *enterica* serotype Reading. Only isolates that met the following criteria were considered: (i) was collected within the United States, (ii) had a known isolation year, and (iii) had a known isolation source. Raw sequencing reads of all identified isolates (*n* = 989) were downloaded from the SRA using the SRA Toolkit (v2.8.2). The majority of animal and retail meat isolates were isolated as a part of U.S. Food Safety and Inspection Service (FSIS) monitoring and the U.S. Food and Drug Administration’s National Antimicrobial Resistance Monitoring System (NARMS) programs. An additional 32 isolates collected from U.S. commercial turkey production facilities were sequenced for this study (see “Sample collection and DNA sequencing” above for details). A series of quality filtering steps within the bioinformatic processing pipeline (described below) were used to obtain a final sample size of 988 high-quality isolate genomes, including 566 from turkey-related sources (see Dataset S3A posted at https://doi.org/10.6084/m9.figshare.11966550). A summary of sample filtering steps is depicted in Fig. S11 posted at the above URL.

To investigate the two recent North American *S*. Reading outbreaks in the context of turkey-source *S*. Reading strains, raw sequencing reads from an additional 111 clinical *S*. Reading isolates collected by the Public Health Agency of Canada’s (PHAC) National Microbiology Laboratory were downloaded from the SRA (see Dataset S3B at the above URL). U.S. and Canada clinical isolates were defined as part of the 2017 − 2019 outbreaks based on criteria that included analysis by whole-genome sequencing defined by the CDC and PHAC, respectively.

### Genome assembly and quality assessment.

All raw FASTQ files were trimmed and quality filtered using Trimmomatic (v0.33) (47), specifying removal of Illumina Nextera adapters, a sliding window of 4 with an average Phred quality score of 20, and 36 as the minimum read length. Trimmed reads were *de novo* assembled using the Shovill pipeline (v1.0.4), which utilizes the SPAdes assembler (48), with default parameters (https://github.com/tseemann/shovill). Assembly quality was assessed with QUAST (v5.0.0) (49). To calculate average sequencing depth of coverage, trimmed reads were mapped to assembled contigs using the BWA-MEM algorithm (v0.7.17) (50), and a histogram of depth was computed using the *genomecov* command in BEDTools (v2.27.1) (51). Only isolates with an *N*50 of ≥20,000 bp and an average depth of ≥20× were included in further analyses (see Fig. S11 posted at https://doi.org/10.6084/m9.figshare.11966550).

### Serotype prediction.

*In silico* serotype prediction was performed with the *Salmonella In Silico* Typing Resource (SISTR) (v1.0.2) (52). Only isolates with a predicted serotype of Reading for both antigen identification and cgMLST cluster analysis were included in downstream analyses (see Fig. S11 posted at the above URL).

### Sequence typing.

*In silico* multilocus sequence typing (MLST) was performed using the software, mlst (v2.16.1) (https://github.com/tseemann/mlst), with the Achtman seven-gene *Salmonella* MLST scheme hosted on the PubMLST website (https://pubmlst.org) (25). Core genome multilocus sequence typing (cgMLST) was performed on the EnteroBase webserver using their custom *Salmonella* cgMLST V2 scheme of 3,002 loci (53). Because draft genomes of multiple contigs may frequently contain missing genes, cgMLST profiles were hierarchically clustered allowing for a mismatch of up to two or five alleles. Minimum spanning trees based on both the traditional MLST and cgMLST allelic profiles were generated in EnteroBase’s standalone software, GrapeTree (v1.5.0) (54).

### Phylogenetic analysis.

Single nucleotide polymorphisms (SNPs) were identified in each sample using Snippy (v4.4.0), with a minimum sequencing depth of 8× (https://github.com/tseemann/snippy) and the *S*. Reading assembly, strain SRR6374143, as the reference. Separate core SNP alignments were then created for all isolates (*n* = 988) and for all clade 1 turkey-source isolates (*n* = 565). Based on MLST and cgMLST minimum spanning trees, one turkey isolate clustered separately from all other turkey isolates and was therefore not included in the turkey-source alignment. Recombinant regions were identified with Gubbins (v2.3.4) (55) and masked from the core genome alignments using maskrc-svg (v0.5) (https://github.com/kwongj/maskrc-svg). Samples with >25% missing data were removed from further analyses (see Fig. S11 posted at https://doi.org/10.6084/m9.figshare.11966550). The program snp-sites (v2.4.1) was then used to extract all core SNPs and monomorphic sites where the columns did not contain any gaps or ambiguous bases (56). Pairwise core SNP distance matrices were created using snp-dists (v0.6.3) (https://github.com/tseemann/snp-dists) after duplicate core SNP profiles were removed with SeqKit (v0.10.1) (57).

Maximum likelihood trees for both all isolates and the turkey-source-only isolates only were reconstructed based on the alignments of core SNPs plus monomorphic sites with IQ-TREE (v1.6.10) (58). ModelFinder was used to identify the most appropriate substitution models according to the Bayesian information criterion (59). For the “all-isolate” tree, the model with the best fit was the three substitution-type model (K3Pu) (60) with empirically derived unequal base frequencies (+F) and the discrete gamma model of rate heterogeneity model with four rate categories (+G4) (61). For the “turkey-source” tree, the best model was K3Pu+F+I, where the rate heterogeneity model (+I) allowed for a proportion of invariable sites. Branch support for both trees was estimated by performing 1,000 ultrafast bootstrap approximation replicates (see Fig. S3 posted at https://doi.org/10.6084/m9.figshare.11966550) (62). The resulting trees were visualized and annotated using the online tool iTOL (63).

To assess the robustness of clades identified in the turkey-source core SNP-based phylogenetic tree, two additional turkey-source trees were constructed using alternative methods based on the pan-genome (see “Pan-genome analyses” below for further details). First, a core genome phylogenetic tree was constructed from the core genome alignment. Core SNPs and monomorphic sites were then extracted from this alignment and used as input into ModelFinder and IQ-TREE. The best model was the transversion substitution model [AG = CT] (TVM) with empirically derived unequal base frequencies (+F) and allowing for a proportion of invariable sites (+I). Branch support was estimated from 1,000 ultrafast bootstrap approximation replicates. Second, a hierarchical clustering dendrogram was generated based on the presence/absence of pan-genome gene clusters. Euclidean distance was calculated using the R package, vegan (v2.5-5) (64), and complete linkage clustering was performed by the *hclust* function from the R package, stats (v3.6.1).

A separate maximum likelihood tree of all clade 1 turkey-source isolates (*n* = 565) and human-source isolates identified as part of the 2017 − 2019 *S*. Reading outbreaks in the United States (*n* = 139) and Canada (*n* = 111) was constructed following the same methods outlined above. As with the turkey-only tree, the best model was identified as K3Pu+F+I, with 1,000 ultrafast bootstrap approximation replicates to estimate branch support.

### Time-scaled phylogenetic analysis.

Nonduplicate turkey-origin isolates were used. A “temporal signal” of the data was evaluated by generating a linear regression of phylogenetic root-to-tip distances against the sampling dates using Tempest (v1.5) (65), and a positive correlation between root-to-tip distance and collection time (*R*^2^ = 0.46) was demonstrated. In addition, the “temporal signal” was verified using a tip-date randomization test that was conducted using the package TipDatingBeast (v1.0.6) (66) in R (v3.4.3) (67). The evaluated TMRCA for the selected model (below) was compared between the real data and the randomized trials (*n* = 20), and no overlaps were found between the HPD_95_ intervals and/or mean values (data not shown). A time-scaled phylogeny was constructed using BEAST (v 1.10.4) (68). A general time reversible (GTR) substitution model was used for nucleotide substitution and both “uncorrelated lognormal relaxed” and “strict” molecular clocks with different coalescent population models (i.e., constant growth, logistic growth, exponential growth, Gaussian Markov random field [GMRF] Bayesian skyride, and Bayesian skyline) were explored. In order to correct for ascertainment bias, the total number of each nucleotide in the reference genome (A, C, G, and T: 1,072,006, 1,166,842, 1,187,745, and 1,074,348, respectively) was manually incorporated in the xml files of all models. Log marginal likelihoods obtained using path sampling (PS)/stepping-stone sampling (SS) (69, 70) were compared. An evolutionary rate of 2.64 × 10^−7^ mutations per site per year, previously estimated for *S*. I 4,[5],12:i:− ST34 (E. Elnekave, S. L. Hong, S. Lim, D. Boxrud, A. Rovira, A. E. Mather, A. Perez, and J. Alvarez, unpublished data) was used as the mean estimation for the clock rate prior. Each model combination was tested for at least two independent Markov chain Monte Carlo (MCMC) runs of at least 200 million generations, with sampling every 20,000 generations. Convergence and proper mixing of all MCMC runs (effective sample size >200) and the agreement between two independent MCMC runs of the same model were verified manually in Tracer (v1.7.1) (71) after excluding 10% of the MCMC chain as a burn-in. The model with the highest log Bayes factor value was the GTR-uncorrelated lognormal relaxed-constant population growth combination. LogCombiner (v1.10.4) (68) was used to combine the two independent MCMC runs of the final model after exclusion of 10% burn-in period. The R package ggtree (v1.10.5) (72) was used for tree visualization.

### Genetic feature identification.

Acquired resistance genes and known chromosomal mutations conferring antibiotic resistance were identified in sample assemblies using staramr (v0.3.0) (https://github.com/phac-nml/staramr) with the ResFinder and PointFinder databases (73, 74). A minimum identity of 90% was used for matching to both databases, with default minimum coverage lengths of 60% for ResFinder and 95% for PointFinder. Plasmid replicon markers were identified using ABRicate (v.0.8.13) (https://github.com/tseemann/abricate) with the PlasmidFinder database (75) and a minimum identity of 90% and minimum coverage length of 60%. ABRicate was also used to screen sample assemblies for the two additional plasmid replicons, Col440II-like and ColRNAI-like (https://github.com/StaPH-B/resistanceDetectionCDC), as they were of interest, but not present in the PlasmidFinder database. A heatmap of the presence and absence of plasmid types and antimicrobial resistance genes was created with the R packages, ggtree (v1.16.4) and tidytree (v0.2.5) (72). To test for significant nonrandom associations between genomic features of interest, one-sided Fisher’s exact tests were performed on 2 × 2 contingency tables using the R function, *fisher.test*, with the Benjamini-Hochberg (BH) procedure to adjust *P* values for multiple testing (76).

### Plasmid and accessory element annotation and analysis.

Based upon plasmid replicon results, two plasmids were selected belonging to IncQ1 and Col440II/RNAI-like replicons. These completed plasmids were searched via nucleotide BLAST across several isolates within each clade to confirm their conservation. Representative plasmid sequences were used from strain SRR8925563. Genes were predicted using Prokka (v1.13.4) (77), and plasmids were annotated and visualized via CLC Sequence Viewer (v8.0.0) (Qiagen, Aarhus, Denmark). For clade-to-clade chromosome comparisons, representative genome assemblies were retrieved for the historical subclade of clade 1 (SRR1583085), contemporary subclade of clade 1 (SRR2407706), emergent subclade of clade 1 (SRR6904571), and clade 3 (SRR5865228) and annotated via Prokka. MAUVE (78) was used to reorder chromosomal contigs of the draft assemblies to that of a completed *S*. Reading chromosome (GenBank accession no. CP030214) (79). MAUVE was then used to align representative chromosomes and compare for genomic differences.

### Plasmid prevalence over time and serotypes.

To determine the prevalence of the Col440II/ColRNAI-like plasmid in Salmonella enterica over time, a 282-bp region of the Col440II-like replicon was used to search the publicly available ENA and SRA databases (through December 2016; 90% identity threshold) (80). Metadata for sequences positive for the 282-bp target were downloaded from NCBI. Resistance gene content was determined using an in-house database adapted from ResFinder 3.0 (90% identity, 60% coverage cutoff). Sequenced isolates with both serotype and year of collection available were included in the analysis (*n* = 100).

### Pan-genome analyses.

Sample assemblies were annotated with Prokka, and a core genome alignment was generated using Roary (v3.12.0) (81). Coding sequences were clustered into “gene clusters” using the default 95% sequence identity. “Core genes” were defined as gene clusters identified in 100% of isolates, while an “accessory genes” were defined as clusters present in <100% of isolates. A presence/absence matrix heatmap of accessory genes was created using the *roary_plots.py* script (https://github.com/sanger-pathogens/Roary/tree/master/contrib/roary_plots). Scoary (v1.6.16) (82) was then used to conduct a pan-genome-wide association analysis comparing the prevalence of gene clusters between phylogenetic clades. Specifically, in the all-isolate trees, clade 1 isolates were compared to clade 3 isolates, and in the turkey-source tree, contemporary subclade isolates were compared separately to both emergent subclade and historical subclade isolates. Genes identically distributed across samples were collapsed into a single gene cluster with the *collapse* option. For the turkey-only tree, a gene cluster was reported as significantly associated with a particular subclade if it had a Benjamini-Hochberg (BH)-adjusted *P* value of ≤0.05 and was present in ≥60% of isolates in one subclade and ≤40% in the other subclade. The reference sequence(s) of each significant gene cluster were then annotated using the top hit(s) from a BLASTX search against the NCBI’s nonredundant protein sequence database (80). Heatmaps comparing the percentage of genomes possessing the significant gene cluster between clades were created using the R package, ggplot2 (v3.2.0) (83). Because not all plasmid replicons of interest were identified by Prokka and thus were not included in the pan-genome analysis, separate 2 × 2 Fisher’s exact tests were performed for each identified plasmid replicon with BH-adjusted *P* values. Follow-up annotations of bacteriophage regions in the *S*. Reading genome were conducted on a representative genome assembly from the contemporary subclade, SRR2407706, with the web-based phage search tool, PHASTER (84).

### Data availability.

Raw reads from isolates sequenced in this study are available at the NCBI Short Read Archive (SRA) under BioProject accession no. PRJNA601793. Supplemental data are available at https://doi.org/10.6084/m9.figshare.11966550.
